# 90-yttrium-ibritumomab tiuxetan as first-line treatment for follicular lymphoma: updated efficacy and safety results at an extended median follow-up of 9.6 years

**DOI:** 10.1007/s00277-022-04781-3

**Published:** 2022-02-12

**Authors:** Kathrin Rieger, Rosaria De Filippi, Ola Lindén, Andreas Viardot, Georg Hess, Kristina Lerch, Peter Neumeister, Andrea Stroux, Caroline A. Peuker, Antonio Pezzutto, Antonello Pinto, Ulrich Keller, Christian W. Scholz

**Affiliations:** 1 Department of Hematology, Oncology and Cancer Immunology, corporate member of Freie Universität Berlin and Humboldt-Universität zu Berlin, Charité – Universitätsmedizin Berlin, Hindenburgdamm 30, 12203 Berlin, Germany; 2 Department of Clinical Medicine and Surgery, Universita degli Studi di Napoli Federico II, Naples, Italy; 3 National Cancer Institute, Fondazione ‘G. Pascale‘, IRCCS, Naples, Italy; 4 Lund University Hospital, Lund, Sweden; 5 Department of Internal Medicine III, University Ulm, Ulm, Germany; 6 Johannes-Gutenberg-University, Mainz, Germany; 7 Medical University Graz, Graz, Austria; 8 Institute for Biometry and Clinical Epidemiology, Charité-Universitätsmedizin Berlin, Berlin, Germany; 9 Internal Medicine III, Technische Universität München, Munich, Germany; 10 German Cancer Consortium (DKTK), partner site Charité – Universitätsmedizin Berlin and German Cancer Research Center (DKFZ), Heidelberg, Germany; 11 Max-Delbrück-Center for Molecular Medicine in the Helmholtz Association, Berlin, Germany; 12 Vivantes Klinikum Am Urban, Berlin, Germany

**Keywords:** Follicular lymphoma, First-line therapy, 90-yttrium-ibritumomab tiuxetan, 90Y-IT

## Abstract

Radioimmunotherapy with 90-yttrium-ibritumomab tiuxetan (90Y-IT) as first-line treatment in patients with follicular lymphoma (FL) demonstrated promising results with a complete remission (CR) rate of 56% and a median progression-free survival (PFS) of 26 months, when initially analyzed after a median follow-up of 30.6 months. The aim of this long-term follow-up was to investigate whether clinical benefits were maintained and new safety signals appeared. Fifty-nine patients, aged ≥ 50 years, with FL grade 1 to 3A in stages II to IV were treated with 90Y-IT as first-line therapy. If CR without evidence of minimal residual disease (MRD), partial response or stable disease was achieved 6 months after treatment, patients were observed without further treatment. Patients with CR but persisting MRD received consolidation therapy with rituximab. The primary endpoint was the clinical response rate. Secondary endpoints were time to progression, safety, and tolerability. After a median follow-up of 9.6 years, median PFS was 3.6 years, and 8-year PFS was 38.3%. Median overall survival (OS) was not reached during the extended follow-up, and 8-year OS amounted to 69.2%. Age 65 years and above or disease progression within 24 months of treatment were significantly associated with shorter OS. An important finding was the lack of new safety signals. In particular, no increase in secondary malignancies or transformation into aggressive lymphoma was observed compared to trials with a similar follow-up. In summary, 90Y-IT as first-line treatment demonstrates a favorable safety profile and long-term clinical activity in a substantial fraction of FL patients in need of therapy. ClinicalTrials.gov Identifier: NCT00772655.

## Introduction

Follicular lymphoma (FL) is an indolent non-Hodgkin lymphoma, frequently diagnosed at an advanced stage, i.e., Ann Arbor stage III or IV. Only about 15 to 25% of the cases are diagnosed at stage I or II [1]. Patients with stage III and IV and low tumor burden according to GELF (Groupe d' Etudes des Lymphomes Folliculaires) criteria are generally only observed (watchful waiting) [2, 3], while individuals with advanced disease and high tumor burden and/or symptomatic lymphoma receive chemoimmunotherapy, i.e., bendamustine or CHOP (cyclophosphamide, doxorubicin, vincristine, prednisone) or CVP (cyclophosphamide, vincristine, prednisone) [4, 5] in combination with the anti-CD20 antibodies rituximab (R) or obinutuzumab (O) [6]. R or O maintenance therapy has been approved and is frequently applied [7–9]. Although FL is a highly radiosensitive disease, external beam radiotherapy (EBRT) is not used upfront in advanced disease and is only infrequently applied in later treatment lines, mainly for palliative purposes. In stage I, or limited stage II FL, EBRT is applied as a curative treatment approach at the time of diagnosis [3]. Recent data from two phase II trials indicate that the addition of R to EBRT increases progression-free survival (PFS) [10, 11]. Radioimmunotherapy (RIT) combines treatment modalities of immuno- and radiotherapy. The radionucleotide 90-Yttrium linked to the anti-CD20 antibody ibritumomab through the linker tiuxetan (90Y-IT, Zevalin®) has demonstrated efficacy as consolidation therapy after first-line chemotherapy [12–15] and in relapsed FL [15]. Side effects of 90Y-IT include in particular neutro-, lympho-, and thrombocytopenia between weeks 6 and 9 after application and are generally well manageable. Severe infections and the need for transfusions are rare if RIT is used in early treatment lines in the absence of severe bone marrow infiltration. Based on these notions, we conducted the first phase II study with 90Y-IT as a stand-alone upfront treatment for patients with advanced stage FL who required treatment. In our first analysis after a median follow-up of 30 months, the overall response rate was 82% (complete response (CR)/CR unconfirmed (CRu) 56%) and median PFS was 26 months [16]. Here, we present updated efficacy and safety results after an extended median follow-up of 9.6 years.

## Methods

### Patients

Patient characteristics have been described in detail before [16] and are summarized in Table 1. Briefly, patients with untreated, histologically confirmed FL grade 1 to 3A in stages III to IV were included. Individuals with stage II FL were included if lesions would have required an extensive radiation field, i.e., an abdominal bath or similar radiation fields that were considered to be unfeasible by the investigator. However, this was not further specified in the protocol. Disease manifestations had to be measurable bidimensionally and patients had to have treatment indication as defined by one of the following criteria: presence of B symptoms, lymphoma progression > 50% within 6 months, organ compression caused by lymphoma lesions, bulky disease (> 5 cm in at least one axis), or FL grade 3A. Due to safety concerns expressed by the local radiation safety authority, recruitment into the trial was limited to patients ≥ 50 years. Patients were ineligible for the trial, if one of the following situations was present: bone marrow infiltration by FL > 25%, peripheral blood (pB) leukopenia (white blood cell count < 2500/μl), thrombocytopenia (platelets < 100,000/μl), circulating lymphoma cells in pB > 500/μl, pleural effusion, ascites > 1000 ml, bulky disease > 10 cm in one axis, or central nervous system (CNS) involvement.

**Table 1:** Patient characteristics at baseline

Baseline characteristics	No. of patients (*n* = 59)	%
Age at assignment, years		
Median	66.0	
Range	51–83	
Gender		
Females	35	59%
Males	24	41%
ECOG performance score		
0	45	76%
1	14	24%
Time from initial diagnosis, months		
Median	2,0	
Range	0–70	
Ann Arbor classification, stage		
I	0	
II	12	20%
III	26	44%
IV	21	36%
Bulky disease at least 5 cm	18	31%
Bone marrow infiltration		
0%	37	63%
1–10%	6	10%
11–25%	16	27%
Grade REAL/WHO		
1	22	37%
2	22	37%
2/3a	3	5%
3a	11	19%
Not classified	1	2%
LDH > upper limit of normal	15	25%
FLIPI score		
Low (≤ 1)	18	31%
Intermediate (2)	25	42%
High (> 2)	16	27%

Percentages are based on patients treated (*n* = 59) unless otherwise indicated

*ECOG*, Eastern Cooperative Oncology Group; *FLIPI*, Follicular Lymphoma International Prognostic Index; *LDH*, lactate dehydrogenase; *REAL*, Revised European-American Lymphoma [classification]; *WHO*, World Health Organization [classification of lymphoid neoplasias 2016]

### Study design and treatment

For this prospective, international, multicenter, non-randomized phase II study (NCT00772655) institutional review board approval from each center and informed consent from each patient were obtained. Patients received R 250 mg/m^2^ on day 1 followed by 185 MBq 111indium for dosimetry. Subsequently, a second infusion of R 250 mg/m^2^ followed by 15 MBq/kg 90Y-IT up to a maximum dose of 1200 MBq was administered on day 8 or 9. Response to treatment according to standard criteria [17] was assessed by computed tomography (CT) scan of the neck, thorax, abdomen, and pelvis every 6 months for 2 years and then once a year for additional 3 years. Patients who achieved a CR but showed evidence of molecular minimal residual disease (MRD) in pB or bone marrow received consolidation therapy with R 375 mg/m^2^ once per week for 4 weeks, followed by 4 cycles of R 375 mg/m^2^ every 8 weeks. Safety and tolerability of treatment were assessed according to Common Terminology Criteria for Adverse Events version 2.0 (CTCAE v2.0). After 5 years of follow-up, patients formally completed the study and were managed according to local guidelines.

### Statistical analysis

The median and interquartile range were calculated for quantitative variables, as well as absolute and relative frequencies for categorical variables. PFS and OS were analyzed using the log-rank test. Results are shown as Kaplan–Meier plots. Cumulative incidences of secondary malignancies and transformation into aggressive lymphoma were estimated according to the Kaplan–Meier method. Cox regression analysis with forward and backward selection was performed to assess the effects of distinct baseline parameters that were significant in univariate analyses, e.g., age, gender, and LDH on PFS and OS. The influence of disease progression within 24 months (POD24) of treatment with 90Y-IT on OS was analyzed with a Cox proportional hazards model as a time-dependent covariate. In our study, POD24 is defined as relapse or disease progression within 24 months after initiation of treatment (modified definition) [18, 19], not after initial diagnosis (original definition). All statistical analyses were performed with the software PASW (IBM SPSS, Chicago, IL). Two-sided *P*-values ≤ 0.05 were considered significant. No Bonferroni adjustment was performed due to the exploratory character of this study.

## Results

### Patient characteristics

Between June 2007 and June 2010, 59 patients with histologically confirmed FL in need of treatment were included in the trial. All 59 patients were treated with 90Y-IT. Only 1 patient received R consolidation therapy subsequently to 90Y-IT according to study protocol. The median age at baseline was 66 years (range 51–83). 17 patients were aged > 70 years and 9 patients > 75 years at the time of treatment. Twelve patients had stage II disease and would have required extended field radiation, while 47 patients were either in stage III (*n* = 26) or stage IV (*n* = 21). Fourteen patients had grade 2-3A (*n* = 3) or grade 3A (*n* = 11) FL, while 45 individuals had FL grade 1–2 (Table 1). All patients fulfilled the treatment criteria as described above. If the GELF criteria had been applied to define high tumor burden, only 2 patients would not have qualified for treatment within the trial due to low tumor burden.

### Clinical response and survival

Of the 59 treated patients, 47 were evaluable for response analysis at 24 months after treatment with 90Y-IT. Of these, 19 (40.4%) patients were in CR at the time, 3 in partial remission (6.4%), and 1 (2.1%) patient had stable disease, whereas disease progression within 24 months after treatment (POD24) was observed in 24 patients (51.1%). Intriguingly, of the 19 patients in CR 24 months after 90Y-IT, 12 patients (63.2%) remained in remission after a median follow-up of 9.6 years, i.e., 20% of all patients treated with 90Y-IT in this trial.

**Fig. 1:**
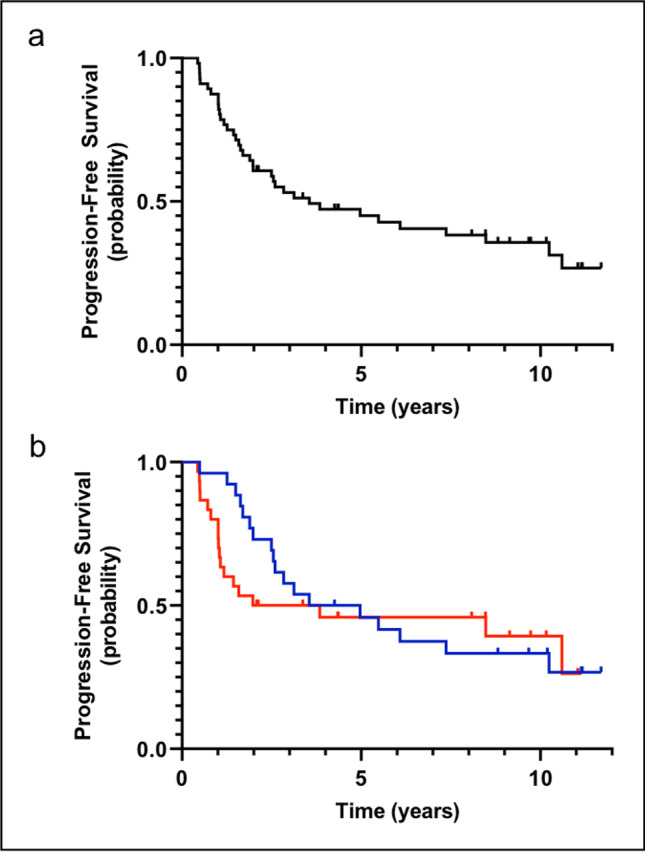
Progression-free survival. **a** Progression-free survival after a median follow-up of 9.6 years among 59 patients treated. **b** Progression-free survival of patients aged 50–64 years (blue curve) as compared to patients ≥ 65 years (red curve) (*P* = 0.607)

Median PFS for the entire patient cohort was 3.6 years (95% CI 0.42–6.68) (Fig. 1A). Eight-year PFS was 38.3%. As in our initial publication [16], in the extended follow-up cohort patients with elevated LDH levels at baseline had a significantly shorter PFS (*P* = 0.046) compared to those with normal levels. In contrast, gender did not have a significant impact on PFS (*P* = 0.552). Furthermore, there was no significant difference regarding PFS between patients ≥ 65 years at baseline as compared to individuals aged 50 to 64 years (*P* = 0.607) (Fig. 1B). A similar result was observed when 60 years (*P* = 0.676) were chosen as a cutoff or when age was applied as a constant variable in a Cox regression model (*P* = 0.606).

**Fig. 2:**
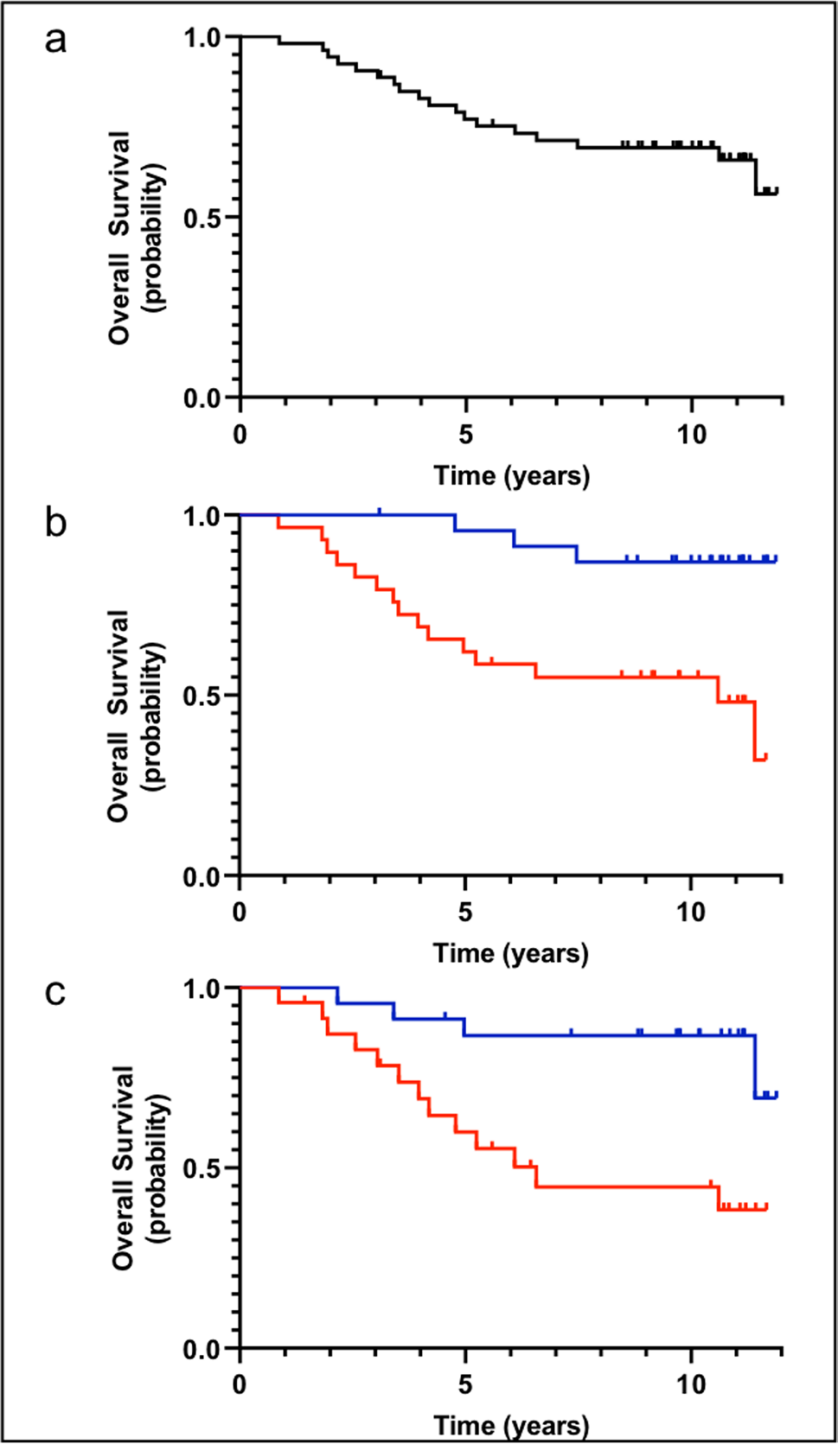
Overall survival. **a** Overall survival after a median follow-up of 9.6 years among 59 patients treated. **b** Overall survival of patients aged 50–64 years (blue curve) as compared to patients ≥ 65 years (red curve) (*P* = 0.002). **c** Overall survival of patients with disease progression within 24 months of 90Y-IT treatment (POD24) (red curve) as compared to patients without disease progression within 24 months (blue curve) (*P* = 0.004) in 47 evaluable patients

Median OS of the cohort has not been reached yet at the time of analysis and 8-year OS was 69.2% (Fig. 2A). Patients with elevated LDH levels at baseline did not have a significantly shorter OS as compared to patients with normal LDH levels (*P* = 0.498). A similar result was observed when LDH was analyzed as a continuous variable in a Cox regression model (*P* = 0.270). Furthermore, gender had no impact on OS (*P* = 0.552). Likewise, being in CR 24 months after treatment with 90Y-IT as compared to not being in CR did not have a significant impact on OS (*P* = 0.114). The same applied for CR at 6, 12, and 18 months, i.e., *P* = 0.893, *P* = 0.246, and *P* = 0.087, respectively. However, patients who were 65 years or older at the time of 90Y-IT application had a significantly higher risk to die during follow-up compared to patients aged 50–64 years (*P* = 0.002) (Fig. 2B). Age applied as a continuous variable in a Cox regression analysis demonstrated a significant impact on OS as well (*P* < 0.001; HR 1.16). Finally, we observed a significantly shorter median OS in patients who experienced disease progression within 24 months after treatment (6.6 years, CI 95% 4.0–9.3) compared to patients who did not relapse within 24 months (median OS not reached during a median follow-up of 9.6 years; log-rank test, *P* = 0.004; hazard ratio for death 4.14 [CI 95% 1.57–10.90]) (Fig. 2C). Remarkably, lymphoma as a cause of death was observed in only 4 out of 15 fatalities indicating the general risk of death in the patient cohort due to relevant comorbidities or secondary cancers in the elderly population.

### Toxicity and safety

Toxicity, which occurred during the first 2 years after treatment, has been reported before [16]. Briefly, CTCAE v2.0 grade 3 or 4 toxicities were exclusively hematologic, i.e., thrombocytopenia (48%), leukopenia (34%), neutropenia (32%), and lymphopenia (20%). Non-hematologic toxicities grade 3 or 4 were not observed.

**Fig. 3:**
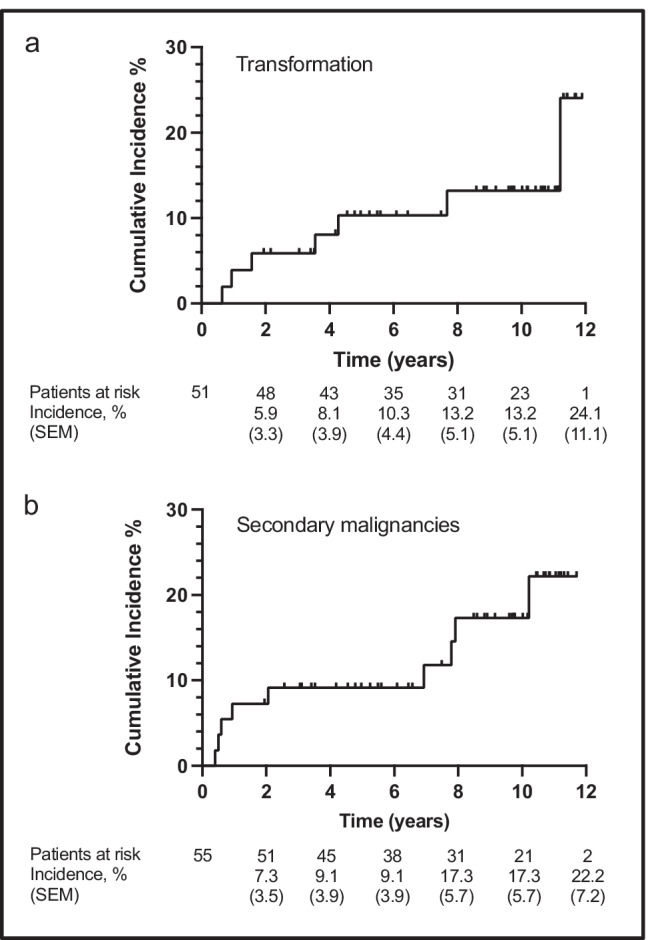
Cumulative incidences. **a** Estimated cumulative incidence of transformation into aggressive lymphoma in 51 evaluable patients. **b** Estimated cumulative incidence of secondary malignancies in 55 evaluable patients. *SEM*, standard error of the mean

With an extended follow-up of 9.6 years transformation into aggressive lymphoma was detected in 7 out of 51 evaluable patients (14%), with a cumulative incidence at 10 years of 13.2% (95% CI 12.2–27.6) (Fig. 3A). Secondary malignancies were observed in 9 out of 55 evaluable patients (16%) including breast cancer, adenocarcinoma of the lung, squamous cell carcinoma of the tongue, cutaneous squamous cell carcinoma, squamous cell carcinoma of head and neck, adenocarcinoma not further specified, CNS neoplasia (no histology obtained), and adenocarcinoma of the esophagus. One patient developed renal cancer as well as pancreatic cancer. Information regarding transformation and secondary malignancies during the prolonged follow-up could not be collected in 8 and 4 patients, respectively. One patient showed no evidence of transformation or secondary malignancy at a shorter follow-up of 5.5 years after treatment. Retrospectively, in two cases of secondary malignancies, signs of the malignant disease were already present before treatment with 90Y-IT. In 3 of the 8 patients, secondary malignancies were fatal. The cumulative incidence of all secondary malignancies at 10 years was 17.3% (95% CI 14.5–25.1) (Fig. 3B).

## Discussion

After a median follow-up of 9.6 years, 90Y-IT application as first-line therapy for FL conferred a median PFS of 3.6 years and an 8-year PFS of 38%. Furthermore, 20% of the 59 patients remain in CR without further lymphoma treatment at the time of this analysis. Our results with an ORR of 87% and a CR/CRu rate of 31% were outperformed to some extent by another trial with 90Y-IT as first-line treatment in FL, demonstrating an ORR of 94%, a CR rate of 86% and an estimated 3-year PFS of 63% [20]. However, only 44% of the patients in that trial had a high tumor burden as defined by GELF criteria, whereas in our trial 96% of the patients would have met GELF criteria, i.e., only 2 of 59 patients did not fulfill GELF criteria. PFS achieved in our trial was comparable to the 8-year event-free survival of 45% reported for treatment-naive patients who received 8 cycles of single-agent R [21]. However, in this trial, patients simply required a single enlarged lymph node ≥ 2 cm for inclusion into the trial, and the proportion of patients with high tumor burden was not disclosed. When comparing our findings to results of chemoimmunotherapies currently used as first-line treatment, i.e., R-CVP, R-CHOP, or R-bendamustine, treatment with 8 cycles of R-CVP let to a median PFS of 32 months in FL patients who required treatment in the opinion of the treating clinician, which was not further specified [4]. In a clinical trial comparing 6 cycles of R-CHOP to R-bendamustine, median PFS was 40.9 months as compared to a median PFS which has not been reached yet after a median follow-up of 45 months (3-year PFS 70%) [22]. In another trial comparing R-CHOP/R-CVP to R-bendamustine, PFS amounted to 40 months as compared to a median PFS which has not been reached yet after a follow-up of 5 years (5-year PFS 58%), respectively [5]. Therefore, PFS achieved with 90Y-IT, i.e., with a single infusion, appears to be comparable to PFS obtained with R-CVP or even R-CHOP.

Apart from analyzing long-term toxicity, the benefit of an extended follow-up is the assessment of OS. Regarding our trial, median OS has not been reached yet after a median follow-up of 9.6 years and 8-year OS was 69%. Notably, POD24 was significantly associated with a shortened OS. While this has been previously demonstrated in FL patients given chemoimmunotherapy [18], more recent studies have highlighted that the predictive impact of POD24 may be prognostic, independent of the type of treatment, and is also applicable to chemotherapy-free regimens [23].

OS demonstrated in our trial is similar to the OS of 68% reported in the trial with R monotherapy after a median follow-up of 9.3 years [21]. However, the median age at trial inclusion was 66 years (range, 51–83) in our trial, while patients in the R monotherapy trial had a median age of 57 years (range, 28–81). The reason for this difference was the prerequisite that patients had to be 50 years or older to participate in our trial, due to the request of the German radiation safety authority. Unfortunately, there is no long-term follow-up data available for the before-mentioned trials examining R-CVP, R-CHOP, and R-bendamustine. However, 8-year OS reported for FL patients treated with a R-CHOP-like regimen, i.e., R-CHVP (cyclophosphamide, doxorubicine, etoposide, prednisolone) plus interferon-α2a amounted to 78% [24]. Notably, the median age in this trial was 61 years (range, 25–75). Regarding results from the PRIMA trial, where FL patients received chemoimmunotherapy (R-CVP, R-CHOP, R-FCM (fludarabine, cyclophosphamide, mitoxantrone)) followed by 12 cycles of R maintenance therapy every 2 months, 10-year OS resulted in 80%, which is superior to OS data from our trial. However, the median age at trial inclusion was only 56 years (range, 22–87) in the PRIMA trial [8]. We demonstrate in our trial that age at study entry had a significant impact on the likelihood of death, and patients > 65 years had a significantly higher risk to die. Therefore, age distribution may be an important factor when putting results from one specific trial in FL patients into a larger clinical context.

Regarding long-term toxicity, we observed 9 secondary malignancies (16%) in 55 evaluable patients during a median follow-up of 9.6 years. If the 2 patients are excluded who retrospectively already showed signs of the malignancy before treatment, the incidence of secondary malignancies would drop to 13% in our trial. This is similar to the R monotherapy trial where 23 secondary malignancies in 151 patients (15%) were reported during a follow-up of 9.3 years [21] or 9% and 7% secondary malignancies following R-CHOP and R-bendamustine therapy, respectively, after a median follow-up of only 45 months [22]. Concerning the lower rate of secondary malignancies in the R-bendamustine trial, the shorter follow-up time and the lower median age of the patients have to be taken into account [22]. The 10-year cumulative incidence of secondary malignancies in our cohort was comparable to recently published data comparing single-dose consolidation therapy with 90Y-IT after R-CHOP with rituximab maintenance therapy in patients with FL [25]. In this trial, 10-year cumulative incidence was 18.5% in 64 patients who received 90Y-IT as consolidation therapy and thus significantly higher than in patients receiving rituximab maintenance therapy (2%). Notably, in our cohort, we exclusively observed epithelial cancers as secondary malignancies rather than hematological malignancies, which would be more expected after 90Y-IT therapy [26]. It cannot be excluded that the considerable high rate of epithelial cancers in our cohort could be attributed, at least in part, to causes unrelated to therapy.

In our trial, transformation into aggressive lymphoma was detected in 7 out of 51 patients (14%), translating into an annual rate of 1.5%, which is in line with published data of 1–3% after immunochemotherapy [27–29].

Our analysis is subject to a number of limitations as this long-term analysis was not preplanned and results were not formally monitored. Furthermore, follow-up procedures were left to the discretion of the treating physician, e.g., regular CT scans were not required as part of the trial after patients had been followed for 5 years.

We have demonstrated that first-line treatment of patients with FL and high tumor burden with 90Y-IT is well manageable and yields a PFS comparable to results achieved with chemoimmunotherapies like R-CVP. Almost two-thirds of the patients achieving CR continue to stay in prolonged remission after 8 years. The extended follow-up did not reveal additional safety signals.

Furthermore, our findings may suggest for the first time that the unfavorable effect on survival of POD24 is retained for FL patients who received 90Y-IT as stand-alone frontline therapy and may prompt including this endpoint in further RIT trials.

In conclusion, after an extended follow-up of more than 9 years, 90Y-IT remains an effective and safe upfront treatment for patients with FL requiring therapy. While availability and application of 90Y-IT have been an issue over the last years, our data support the validity and safety of RIT-based upfront therapy and prompt further trials of novel antibody-radionuclide-conjugates as frontline treatment of FL [30, 31].

## Declarations

### Data sharing statement

Clinical data contains potentially personal information and is stored with the authors. Upon request, it can be shared in anonymized form with the editors or fellow scientists.
